# Electrospraying as a Means of Loading Itraconazole into Mesoporous Silica for Enhanced Dissolution

**DOI:** 10.3390/pharmaceutics16081102

**Published:** 2024-08-22

**Authors:** Charitini Volitaki, Andrew Lewis, Duncan Q. M. Craig, Asma Buanz

**Affiliations:** 1 School of Pharmacy, Faculty of Life Sciences, UCL, 29-39 Brunswick Square, London WC1N 1AX, UK; 2 Quotient Sciences, Mere Way, Ruddington, Nottingham NG11 6JS, UK; 3 Faculty of Life Sciences, University of Bath, Claverton Down, Bath BA2 7AY, UK; dqmc21@bath.ac.uk; 4 School of Science, Faculty of Engineering and Science, University of Greenwich, Gillingham ME4 4TB, UK

**Keywords:** mesoporous silica particles, electrospraying, itraconazole, rotary evaporation, dissolution rate, amorphous

## Abstract

Mesoporous silica particles (MSPs) have been investigated as potential carriers to increase the apparent solubility and dissolution rate of poorly water-soluble drugs by physically stabilising the amorphous nature of the loaded drug. In preparing such systems, it is recognized that the loading method has a critical impact on the physical state and performance of the drug. To date, there has been very limited investigation into the use of electrospraying for loading drugs into mesoporous silica. In this study, we further explore the use of this approach, in particular as a means of producing amorphous and high drug-loaded MSPs; the study includes an investigation of the effect of drug loading and MSP concentration on the formulation performance and process. A comparison with rotary evaporation, a more widely utilised loading technique, was conducted to assess the relative effectiveness of electrospraying. The physical state of the drug in the formulations was assessed using powder X-ray diffraction (PXRD) and differential scanning calorimetry (DSC). The drug release profiles were determined by a comparative in vitro drug release test. Electrospraying successfully produced formulations containing amorphous drug even at a high drug loading. In contrast, while itraconazole was present in amorphous form at the lower drug-loaded formulations produced by rotary evaporation, the drug was in the crystalline state at the higher loadings. The percentage of drug released was enhanced up to ten times compared to that of pure itraconazole for all the formulations apart from the highest loaded (crystalline) formulation prepared by rotary evaporation. Supersaturation for at least six hours was maintained by the formulations loaded with up to 30 mg/mL itraconazole produced by electrospraying. Overall, the results of this study demonstrate that electrospraying is capable of producing amorphous drug-loaded MSPs at high loadings, with associated favourable release characteristics. A comparison with the standard rotary evaporation approach indicates that electrospraying may be more effective for the production of higher loadings of amorphous material.

## 1. Introduction

Oral delivery of drugs is the most common and convenient route of administration. However, many new active pharmaceutical ingredients (APIs) have low solubility, leading to slow dissolution and poor absorption after oral administration [1]. Approaches to improve solubility include chemical modifications such as co-crystal and salt formation, inclusion complexation and physical modifications such as micronisation, formation of nanosuspension, polymeric micelles, solid lipid nanoparticles, polymorph control and stabilizing the drug in the amorphous or molecular form via dispersion in a carrier [2,3]. 

Mesoporous silica particles (MSPs) have been investigated as a potential carrier to increase the apparent solubility and dissolution rate of poorly water-soluble drugs by physically stabilising the loaded drugs in their amorphous state. They have a large surface area that leads to a high surface free energy; the physical stability of the amorphous drugs in MSPs is ascribed to a reduction in the Gibbs free energy of the system due to the adsorption of the drug on the pre surfaces. In addition, it has been suggested that drugs may not recrystallise when present in confined spaces, typically smaller than ten times the size of the drug molecule; thus, the pore size is critical to preventing crystallisation [4]. Therefore, the loaded drug can potentially be physically stabilized in an amorphous or molecularly dispersed state in the mesopores, leading to supersaturation and improved dissolution rates [5]. 

The loading method into the MSPs may have a pivotal effect on the physical state of the drug and, therefore, its dissolution [6]. Also, it will affect the encapsulation efficiency, drug distribution and physicochemical properties [7]. Solvent immersion and solvent impregnation are the two most widely used techniques for the drug loading of MSPs; other methods that have been reported include melt processes, spray drying and loading with supercritical CO_2_. There has been limited investigation into the use of electrospraying for loading drugs into mesoporous silica, one such study being Sayed et al. who used electrospraying as a drug loading technique for mesoporous and non-porous silica particles at a drug loading of 25% *w*/*w* and a lower silica concentration [8]. A high drug loading (≥30%) of MSPs has been reported to lead to the presence of the crystalline drug in studies that used the following loading techniques: solvent evaporation [8,9], wetness impregnation [10,11] and immersion [12]. Here, we investigated electrospraying as a technique to produce amorphous and high drug-loaded MSPs while evaluating the effect of the drug loading and MSP concentrations on the product characteristics and performance. 

Electrospraying has been gaining attention within the drug delivery field as a means of producing amorphous drugs as well as facilitating particle production with a well-defined size and architecture [13]. It is also known as electrohydrodynamic atomization and utilises an electrical field (electro) to manipulate the motion of liquids (hydrodynamic) to achieve the conversion of liquids to fine droplets (atomisation). A basic setup would comprise a syringe pump, a syringe, a metal needle; the emitter, a high-voltage power source and a grounded substrate, the collector [14]. A high voltage is applied to the emitter, resulting in charging the liquid stream, which causes electrostatic stress inside the liquid as it leaves the emitter. The electric forces compete with the surface tension of the droplet, deforming the meniscus to a conical shape, the Taylor cone. Eventually, the electrostatic force overcomes the surface tension, and the excess charge is gathered at the tip of the cone, creating a jet, which further breaks up to droplets. The solvent quickly evaporates during the flight of the droplets towards the collector because of their small size (10^−6^ to 10^−9^ m), leading to a dry solid dispersion. The high surface charge that the sprayed droplets possess leads to Coulombic repulsion, causing self-dispersion of the droplets and preventing agglomeration during drying [13,15]. Consequently, the solubility and dissolution rate of a poorly soluble drug can benefit not only from the amorphization but also from the high surface area available. Compared to conventional methods, electrospraying presents the advantage of producing particles below the micrometre range with a narrow particle size distribution and much less agglomeration due to self-dispersing properties [13]. 

In order to assess the effectiveness of electrospraying to load MSPs, we performed a direct comparison with the more standard rotary evaporation approach [9,16]. The rotary evaporator utilises reduced pressure to lower the solvent boiling point, while rotation of the sample increases the effective surface area and heating increases the rate of solvent evaporation [16]. The simplicity and scalability of the method has allowed for it to become one of the most common techniques for drug loading MSPs [9]. 

Our model drug is itraconazole (ITZ), an antifungal medication that is used to treat infections occurring throughout the body, including the lungs, mouth, throat, toenails or fingernails. ITZ has poor aqueous solubility, estimated at ca. 1 ng/mL at neutral pH and ca. 4 μg/mL at pH 1 because of its high lattice energy and extreme hydrophobic character [8]. The pKa of itraconazole is 3.7, which indicates a very weak basic drug; the LogP is 5.66. 

Mesoporous silica has been used before to enhance the dissolution profile of itraconazole. Mellaerts et al. utilised MSPs of different pore sizes (SBA-15 of 4.5, 6.4, 7.9 and 9 nm) to demonstrate that the presence of a sufficiently wide pore diameter is key to accelerating the release of the drug. Moreover, they suggested the existence of a critical loading to incorporate molecularly dispersed drugs in the SBA-15 materials; loading beyond the critical point leads to the generation of crystalline regions. For example, itraconazole crystallised within the silica particles (SBA-15) at 30% loading using solvent evaporation [8]. MSPs (SBA-15) were loaded with itraconazole and ibuprofen using three different procedures: (i) adsorption from solution, (ii) incipient wetness impregnation and (iii) heating of a mixture of drug and SBA-15 powder. It was revealed that the physical state of hydrophobic drug molecules in MSPs was influenced by the loading procedure [17]. Kinnari et al. used different mesoporous silicon (thermally oxidized and thermally carbonized) and non-ordered mesoporous silica (Syloid AL-1 and 244 FP) microparticles to evaluate the structural effect of the particles on the drug loading degree and on the dissolution behaviour of the itraconazole. The results showed that the surface structure of the mesoporous silica particles containing large amounts of silanol groups, the silica pore structure and the small particle size were advantageous properties for the efficient loading of itraconazole, as was the high pore volume. The physicochemical properties of the particles were the crucial parameters for the drug loading efficiency, the drug release kinetics and the stability of the loaded drug [18].

Overall, therefore, there is some limited evidence that electrospraying is an effective means of loading MSPs [6] and also evidence that MSPs may be an effective means of formulating itraconazole [18,19,20]. This study aimed to achieve three interrelated objectives. Firstly, we wished to further explore the use of electrospraying to load MSPs, in particular examining the effects of parameter control so as to develop a more generalizable knowledge base to allow for further use of this approach. Secondly, we compared electrosprayed MSPs with those prepared using a more conventional approach so as to ascertain the advantages or disadvantages of the electrospraying technique in terms of product characteristics and performance. Finally, we explored the use of the electrospraying approach to the formulation of a relevant model drug, itraconazole, particularly examining the effects of drug loading on product performance. We used Syloid 244FP (Syl) mesoporous silica; this is a non-ordered porous silicon dioxide with a neutral pH and randomly oriented pores with an average pore size of 19 nm in diameter [9]. A successful outcome of this study is an enhanced understanding of using electrospraying as a means of producing drug-loaded mesoporous silica particles for enhanced drug dissolution, gaining in-depth knowledge of the similarities and/or differences between electrospraying and rotary evaporation in the solid state and release profile of the drug from formulations and producing amorphous and high ITZ-loaded MSPs.

## 2. Materials and Methods

### 2.1. Materials

Itraconazole (ITZ) was supplied by Watson Noke Scientific, Kunshan, China. Sylloid 244FP was obtained from W.R. Grace, Columbia, MD, USA. Sporanox was produced by Janseen-Cilag, High Wycombe, UK. Dichloromethane (DCM), ethanol and acetonitrile (ACN) were purchased from Sigma-Aldrich, St. Louis, MO, USA. Phosphoric acid and potassium phosphate monohydrate for the HPLC method were purchased by Honeywell Fluka, Seelze, Germany. Fasted State Simulated Gastric Fluid (FaSSGF) for an in vitro drug release study was purchased from Biorelevant.com, London, UK and it contained physiological surfactants (bile salts and lecithin) present in the gastrointestinal tract to simulate the fluids. The FaSSGF had an acidic pH of 1.6, which facilitates the solubilisation of weak bases such as itraconazole.

### 2.2. Methods

#### 2.2.1. Fabrication of Itraconazole-Loaded Particles

Known amounts of itraconazole were weighed and dissolved in DCM:Ethanol (1:1) under magnetic stirring for one hour to obtain 0.10, 0.25, 0.30 and 0.75% *w*/*v* solutions. Subsequently, suitable amounts of silica at a ratio of 10:1 or 3.3:1 to ITZ were added to the solutions. The suspensions were left under magnetic stirring for 24 h and were sonicated for 10 min before use. The detailed ITZ concentrations and ITZ:Syl ratios are shown in Table 1.

Subsequently, either electrospraying or rotary evaporation was employed. A Spraybase^®^ electrospray apparatus (Spraybase Electrospinning kit by Spraybase, a sub-division of Avectas Ltd., Dublin, Ireland) was used to obtain the electrosprayed ITZ-loaded Syl particles. The applied voltage ranged between 15 and 21 kV; a gap distance of 20 cm, needle of 24 G and flow rate of 0.8 mL/h were the optimised parameters used. A Rotavapor R-300 with B-300 Base by BUCHI, UK was used to obtain the RE ITZ-loaded Syl particles. The solvent was evaporated over for 30 min at 50 °C, 100 RPM and 100 mbar.

#### 2.2.2. Scanning Electron Microscopy (SEM)

The morphology of the particles was assessed by using a scanning electron microscope. The samples were sputter-coated with a thin layer (10 nm) of gold using a Quorum Q150T Sputter Coater (Quorum Technologies Ltd., Laughton, East Sussex, UK) in an argon atmosphere. Subsequently, they were imaged under FEI Quanta 200F (FEI company Ltd., Eindhoven, The Netherlands) at an accelerating voltage of 5 kV. Image analysis was performed using Fiji software (https://imagej.net/software/fiji/downloads, accessed on 24 June 2024) [21].

#### 2.2.3. Nitrogen Sorption Isotherms

Syloid 244FP and the formulations produced by electrospraying were characterized by nitrogen gas desorption/adsorption isotherm measured at −196 °C using a Quantachrome Quadrasorb Evo. Before the physisorption measurements, the samples were degassed at 250 °C under vacuum for 24 h. Total surface area (St) was determined using the Brauner–Emmett–Teller (BET) model over a relative pressure (P/P0) range between 0 and 1. The pore volume and pore size distribution were derived from the density functional theory (DFT) method. Total pore volume was estimated from the amount adsorbed at a relative pressure of 0.99 [22].

#### 2.2.4. Encapsulation Efficiency (EE)

The percentage of itraconazole that was loaded in silica particles was determined with the following procedure: 2 mg (A total of 2 mg of the samples was used, instead of a whole batch, due to the low yield of this technique on the lab-scale. Each batch was characterised (XRD, DSC) and used for a complimentary analysis of the formulation; thus, smaller amounts were used for each analysis. The same amount of particles produced using rotary evaporator was analysed for the determination of the encapsulation efficiency for consistency purposes.) of each formulation was dissolved in 20 mL of acetonitrile under magnetic stirring for 24 h. Then, 10 mL of the resulting suspension was centrifuged at 9900 rpm for 1 h using a Sigma 3-16KL centrifuge. The concentration of itraconazole was then determined using the described HPLC method.

The EE% was calculated using the following equation:

$$EE\%=\frac{Experimental\;Amount}{Theoretical\;Amount}*100$$

The experimental amount is the amount of ITZ detected in acetonitrile using HPLC, while the theoretical amount of the formulation is the amount of itraconazole that should exist in the formulation if 100% of the drug was incorporated.

#### 2.2.5. Powder X-ray Diffraction (PXRD)

Analysis was performed using Miniflex 600 diffractometer (RigaKu, Tokyo, Japan) with CuΚα1 radiation (λ = 1.54006 Å) at 40 kV and 30 mA. The patterns were recorded at room temperature from 3 to 45° at a scanning speed of 2°/min.

#### 2.2.6. Modulated Temperature Differential Scanning Calorimetry (MTDSC)

Thermal analysis was performed using DSC Q2000 (TA instrument, Waters Corporation, Milford, MA, USA). Then, 2–3 mg of each sample was weighed and filled into Tzero pans, which were then sealed with pin-holed hermetic lids to allow for volatiles to escape from the pan. A modulated temperature DSC running with a modulation of ±0.212 °C every 40 s at an underlying heating rate of 2 °C/min was used to heat the samples from 20 °C to 180 °C. Itraconazole was tested using conventional DSC as well using a heat–cool–heat cycle performed from 20 to 180 °C at a ramp of 2 °C/min, equilibrating at 20 °C and heating again up to 180 °C at a ramp of 2 °C/min. All experiments were performed using dry nitrogen as purge gas at a rate of 50 mL/min. The heat flow signal was used to plot the results. The calibration of the temperature and enthalpy of the instrument were performed by heating an indium standard through its melting transition and measuring the temperature and enthalpy of the melting endotherm (Tm = 156.6 °C; the heat of fusion = 28.57 J/g).

#### 2.2.7. Comparative In Vitro Drug Release

The following process was carried out to determine the in vitro drug release profile of formulations (cumulative release %). Amounts of the formulations containing 0.4 mg/mL of itraconazole were weighed in glass vials, and 10 mL of Fasted Simulated Gastric Fluid (FaSSGF) was added to each vial. The vials were incubated at 37 °C and 100 rpm in the Incu Shake MINI. At define time points (1, 2, 4, 6, 24 h), 0.5 mL of the releasing medium was withdrawn. The aliquot was centrifuged for 15 min at 15,000 rpm using Scispin Micro and then filtered through 0.22 μm PTEE Fisherbrand filters. The released amount of itraconazole was quantified using the HPLC method.

#### 2.2.8. High-Performance Liquid Chromatography (HPLC) Method

The concentration of ITZ in the acetonitrile (drug loading assay) and FaSSGF (in vitro drug release) was analysed by the Agilent HPLC system. A Phenomenex Prodigy ODS column (150 mm × 4.6 mm, 5 μm) at 40 °C was used. The mobile phase was composed of 70% acetonitrile and 30% formic acid (0.1%) previously adjusted to pH 3.0. The flow rate was 1.25 mL/min using an isocratic method. The detection UV wavelength was set as 254 nm. The injection volume was 20 µL.

## 3. Results and Discussion

### 3.1. ITZ-Loaded Syloid 244FP Particles Produced by Electrospraying

**Figure 1:**
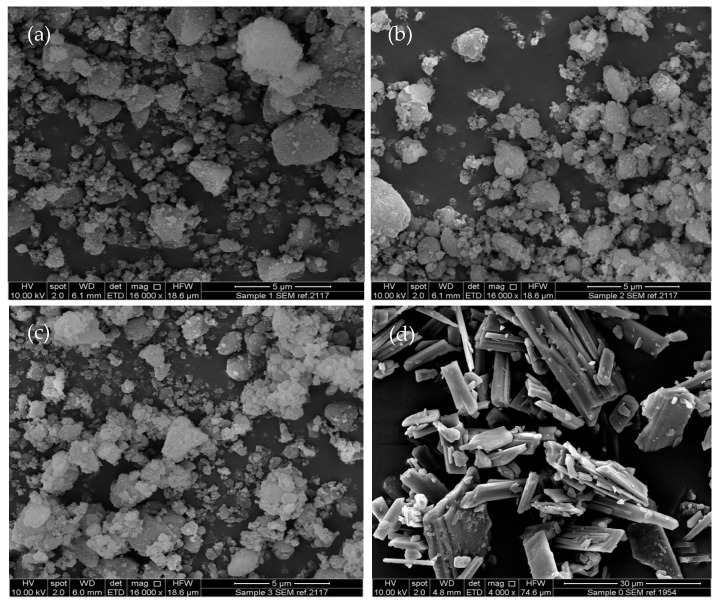
SEM images of (**a**) unprocessed Syloid 244FP, (**b**) unloaded electrosprayed particles of 1% *w*/*v* Syl and (**c**) 2.5% *w*/*v* Syl, (**d**) pure itraconazole.

**Figure 2:**
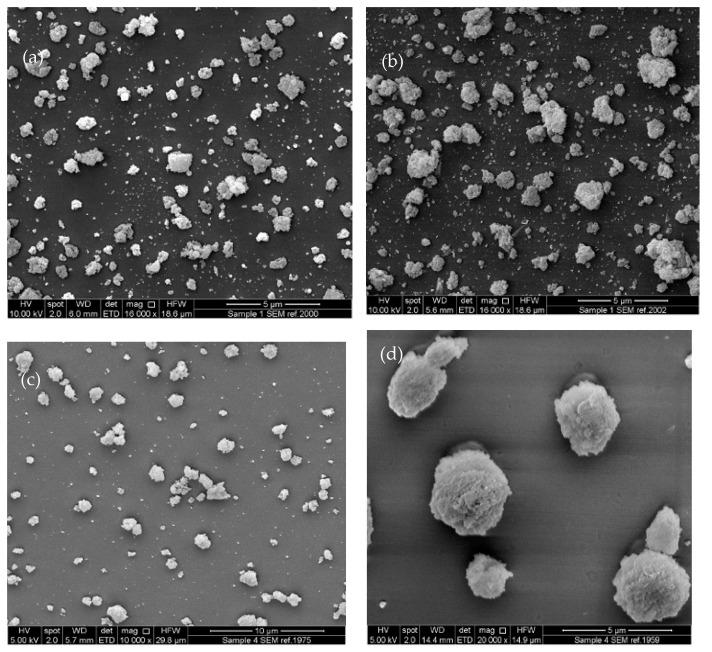
SEM images of (**a**) 1% *w*/*v* Syl 1:10 ITZ:Syl, (**b**) 1% *w*/*v* Syl 1:3.3 ITZ:Syl, (**c**) 2.5% *w*/*v* Syl 1:10 ITZ:Syl, (**d**) 2.5% *w*/*v* Syl 1:3.3 ITZ:Syl produced by electrospraying.

**Figure 3:**
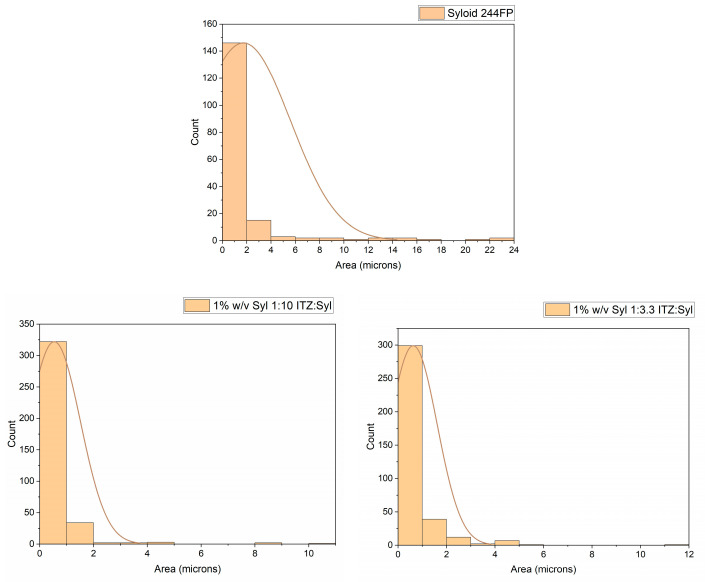
Particle size and particle size distribution of Syloid 244FP, 1% *w*/*v* Syl 1:10 ITZ:Syl and 1% *w*/*v* Syl 1:3.3 ITZ:Syl. The curve depicts the mean size of the particles.

**Figure 4:**
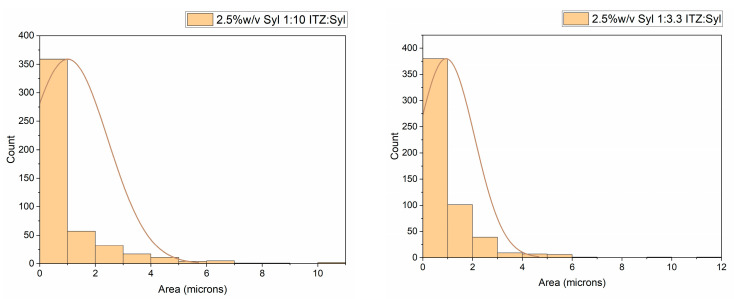
Particle size and particle size distribution of 2.5% *w*/*v* Syl 1:10 ITZ:Syl, 2.5% *w*/*v* Syl 1:3.3 ITZ:Syl. The curve depicts the mean size of the particles.

SEM images of unprocessed Syloid 244FP and unloaded electrosprayed particles of 1% and 2.5% *w*/*v* Syl in DCM:Ethanol (1:1) are shown in Figure 1. ITZ-loaded Sylloid 244FP was successfully produced using electrospraying at higher and lower drug loadings and different Syloid 244FP concentrations. The particles of all formulations were submicron in size, as shown in Figure 2. SEM images of unprocessed itraconazole, Syloid 244FP and unloaded electrosprayed particles of 1% and 2.5% *w*/*v* Syl in DCM:Ethanol (1:1) are shown in Figure 1 for comparative purposes. The SEM image of pure itraconazole presents needle-shape crystals of different sizes with a flat, smooth surface. The image of unprocessed Syloid 244FP depicts a heterogeneous sample with particles of many different sizes. This observation is further supported by the particle size distribution results (Figure 3) showing particles from the submicron size range to 20 μm. The electrosprayed samples were also heterogeneous in size. It was observed that the size, shape and rough surface (morphology) of Syloid 244FP did not change when it was processed using electrospraying nor did it change when it was loaded with itraconazole. The morphology of ITZ-loaded Syl particles remained the same, while they presented a lower particle size variation from the submicron size range to 8 μm (Figure 3 and Figure 4). It is possible that aggregates of unprocessed Syloid 244FP broke down due to the sonication of the ITZ-loaded Syl suspension before electrospraying, leading to a lower particle size variation. It has been previously demonstrated that sonication produces a significant particle size decrease for talc and vermiculite in the submicron range [23,24]. Similar findings were reported by Sayed et al., who used electrospraying to load a novel chalcone in mesoporous and non-porous silica particles. It was stated that particle surface morphology was maintained, showing a surface with little to no drug crystals present. They suggested that most of the drug loaded was encapsulated within the pores of the mesoporous silica [6]. Additionally, Limnell et al., who loaded Syloid 244FP with indomethacin using immersion and solvent evaporation loading methods, reported that the morphology of Syloid 244FP did not change after loading or tablet compression [12].

The particle size and particle size distribution of Syloid 244FP and the formulations were measured from the SEM images of the formulations, and the results are shown in Figure 3 and Figure 4. The majority of the particles of Syloid 244FP were between 0 and 1 μm; however, there were some larger particles of up to 20 μm in size. The majority of the electrosprayed particles of all formulations were from 0 to 1 μm, indicating small-sized particles of a narrow size distribution. However, a wider distribution around the mean size was observed for the 2.5% *w*/*v* Syl formulations. It seemed like drug loading did not affect the particle size.

Nitrogen sorption analysis provides insight into the physical adsorption of gas molecules on solid samples to measure the specific surface area of the material [25]. The surface area and pore volume of Syloid 244FP and formulations prepared using electrospraying were evaluated using nitrogen sorption isotherms. As shown in Table 2*,* the surface area of Syloid 244FP was 364.7 m^2^/g, and the pore volume was 1.74 cm^3^/g. The nitrogen sorption analysis of the formulations revealed a significant reduction in the surface area of ITZ-loaded Syl particles, indicating that itraconazole was present. Moreover, the decrease in the pore volume of the formulations most likely indicates that itraconazole was successfully incorporated into the pores.

Similarly, the reduction in the surface area and pore volume has been used as an indication of successful drug loading in many other studies. Shen et al. used spray-drying to load ibuprofen in MSPSs of different pore and particle sizes at (50% *w*/*w* drug loading) to evaluate the effect of those parameters on the dissolution profile of the drug. The nitrogen adsorption of MCM-41, SBA-15 and SBS-15-LP (SBA-15 with enlarged pores) was measured before and after the loading of ibuprofen. The surface area and pore volume decreased significantly after loading, indicating that most of the pore area was occupied by ibuprofen [26]. Limnell et al. loaded indomethacin in MCM-41 and SBA-15 using an immersion technique (*ca* 40% *w*/*w* drug loading). They reported that nitrogen sorption showed a significant reduction in pore volume in both MSPs due to the incorporation of a high amount of indomethacin [12]. Biswas synthesised MSPS and functionalised MSPs for pH-dependent release and loaded them with valsartan using the solvent evaporation method. The significant decrease in the surface area and pore volume of the MSPs and functionalised MSPs loaded with valsartan was attributed to the successful loading of the drug in the mesopores [27].

**Figure 5:**
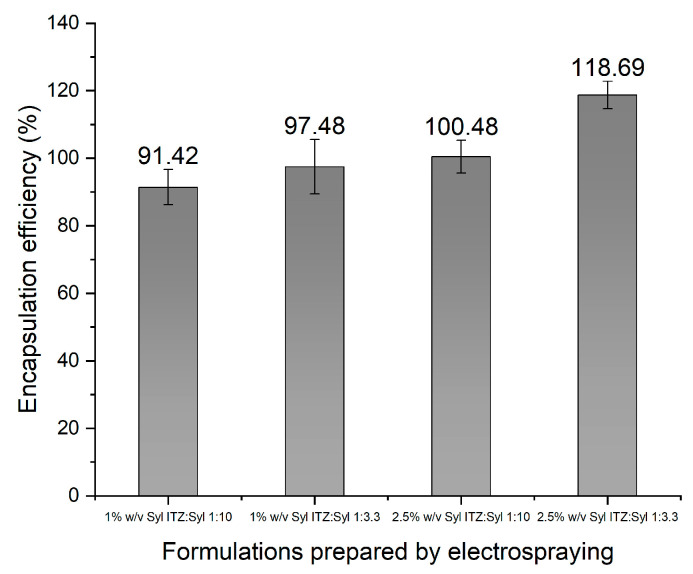
Percentage of encapsulation efficiency of 1% *w*/*v* Syl 1:10 ITZ:Syl, 0.5% *w*/*v* Syl 1:10 ITZ:Syl, 1% *w*/*v* Syl 1:3.3 ITZ:Syl, 2.5% *w*/*v* Syl 1:3.3 ITZ:Syl prepared by electrospraying.

The encapsulation efficiency of all the formulations was close to 100% (Figure 5), indicating that itraconazole was successfully deposited in Syloid 244FP. The nitrogen sorption results suggested that itraconazole was successfully incorporated into the pores, based on the decrease in the pore volume of the formulations. Combining the results of these two analytical techniques, we can conclude that itraconazole is present in the formulations at almost (91–100%) of the initial drug loading, and part of it is incorporated in the pores of Syloid 244FP. The formulation of 2.5% *w*/*v* Syl 1:3.3 ITZ:Syl presented an EE of 118 ± 4.07. This potentially indicates that the experimental amount of itraconazole in the sample was higher than the theoretical amount, leading to an encapsulation efficiency higher than 100%. The theoretical amount was calculated based on the ITZ:Syl ratio of each formulation. An amount of Syloid 244FP was probably removed during electrospraying, leading to an inaccurate theoretical value. Syloid 244FP probably precipitated in the syringe over time; the suspension was left to be electrosprayed for approximately 5 h, forming a thin layer of white silica gel. This resulted in an increased amount of itraconazole in the samples, which could be deposited on the Syloid 244 FP particles or form a bulk phase. Sayed et al. utilised electrospraying to load SBA-15 with a novel chalcone that they synthesized [6]. They reported an encapsulation efficiency higher than 100% (132%) and suggested that the cause was the sedimentation of silica particles in the drug solution during the electrospraying process [6], which is in agreement with our findings.

**Figure 6:**
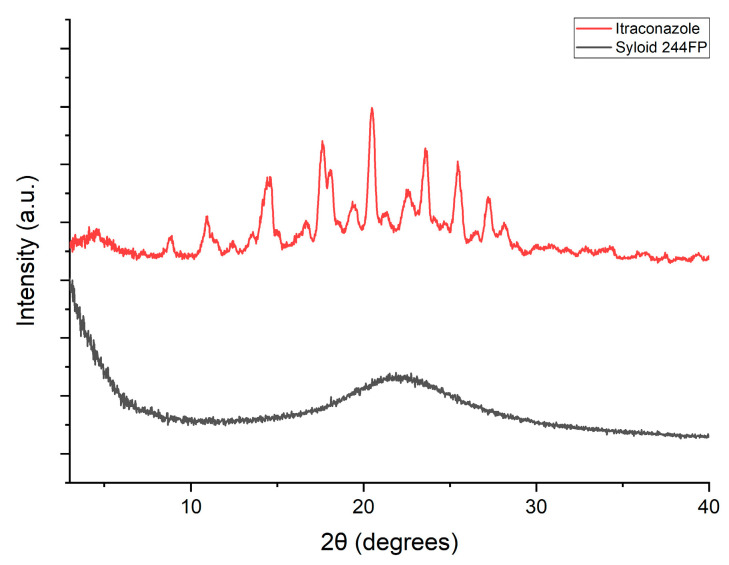
PXRD pattern of itraconazole and Syloid 244FP.

**Figure 7:**
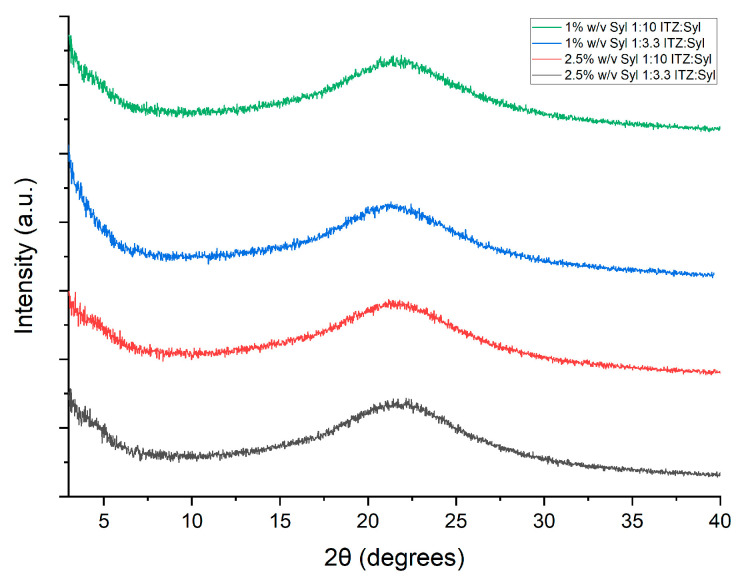
PXRD pattern of the formulations produced by electrospraying (below).

The PXRD pattern of pure itraconazole illustrates peaks at 17.5°, 18°, 20.4° and 23° (Figure 6), which are the typical peaks for itraconazole [28], indicating a crystalline material. The PXRD pattern of Syloid 244FP shows a broad halo without any diffraction peaks from 0 to 40° 2θ degrees (Figure 6), indicating that all the diffraction peaks that are presented in this 2θ-degrees window correspond to itraconazole. A lack of diffraction peaks in the PXRD patterns of all formulations most likely indicates that itraconazole exists in the amorphous state in the formulations of both the higher and lower drug loadings (Figure 7).

**Figure 8:**
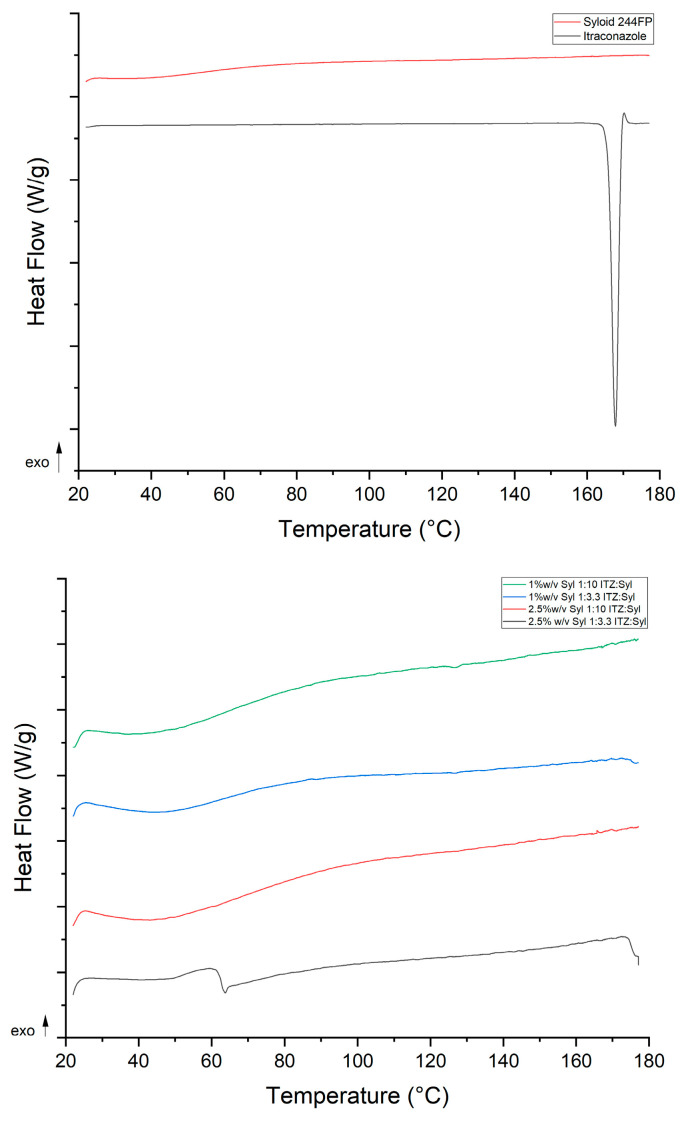
MTDSC traces of itraconazole, Syloid 244FP (**above**) and the formulations produced by electrospraying (**below**) (Total Heat Flow signal is depicted).

The MTDSC trace of unprocessed itraconazole presented a melting point at 168 °C, indicating a crystalline material. Crystalline itraconazole melts at 167 °C, while glassy itraconazole is characterized by three typical endothermic transitions upon heating: a glass transition at 60 °C and two endothermic transitions due to its liquid–crystalline nature at 75 and 90 °C [8]. The Sylloid 244FP trace (Figure 8 above) shows a broad endotherm between 30 and 80 °C, indicating probably water loss as silica is a hygroscopic material [5]. The melting point of silica (All silica forms are made from the same chemicals but can have different structures. Silica is divided into two main groups: crystalline silica and amorphous silica (non-crystalline silica). In crystalline silica, the silicon and oxygen atoms are arranged in a fixed geometric pattern. In contrast, in amorphous silica, no spatial ordering of the atoms is present [29]) is 1710 °C [30] which is a much higher temperature than this protocol covers. MTDSC data of the physical mixtures of ITZ:Syl at ratios 1:3.3 and 1:10 (Figure S1) depicted endothermic peaks at 167 °C, indicating that crystalline itraconazole was present. This shows that MTDSC is sensitive enough to detect the presence of crystalline material at the ratios used to prepare the formulations. The MTDSC data of the electrosprayed formulations presented no endothermic transition peaks of itraconazole (Figure 8 below), again suggesting the molecular dispersion of itraconazole in the formulations. Similar findings were observed by Kinnari et al. who loaded itraconazole using the immersion method in Syloid AL-1, Syloid 244FP, TOPSi and TCPSi at high (33% *w*/*v*) and low (22% *w*/*v*) drug loadings. No transition that could correspond to itraconazole was observed in any of the DSC traces of the formulations, suggesting that itraconazole was molecularly dispersed in the MSPs at both drug loading and all MSPs [18].

All the formulations presented a broad endothermic peak from 30 to 80 °C, similar to that in the trace of Syloid 244FP, suggesting evaporation of solvent or water that is confined in the pores. The MTDSC data of 2.5% *w*/*v* Syl 1:3.3 ITZ:Syl presented a glass transition at 64 °C, confirming that itraconazole exists in an amorphous state or liquid–crystal form. Mellaerts et al. loaded SBA-15 with itraconazole and ibuprofen using solvent evaporation, wetness impregnation and the melt method at drug loadings of 20 and 30% *w*/*w* [17]. The DSC data of the formulations loaded at 20% *w*/*w* prepared by solvent evaporation and wetness impregnation showed no transition of drugs, while the formulation of itraconazole prepared by the melt process at the same drug loading showed an endothermic peak corresponding to the melting point of itraconazole. Endothermic transitions corresponding to glass transitions of itraconazole were observed for the formulation of itraconazole prepared at 30% *w*/*w* by all methods. A melting point was present as well at the trace of the formulation of itraconazole prepared by the melt method at 30% *w*/*w*. The author suggested that the glass transition revealed the formation of an itraconazole bulk phase [17]. Similarly, the glass transition at the trace of 2.5% *w*/*v* Syloid 244FP at ITZ:Syl 1:3.3 probably indicates the formation of a bulk phase of amorphous itraconazole. All the MTDSC results were in agreement with the PXRD results, confirming the amorphous state of itraconazole in the formulations.

Comparative drug release testing was performed for itraconazole, Sporanox (the commercially available product of itraconazole) and the electrosprayed formulations in triplicates for 24 h in FaSSGF in order to evaluate the effect of the drug loading on the release profile of itraconazole. Sporanox is a capsule-based formulation containing sugar spheres coated with an itraconazole solid dispersion in hydroxypropylmethyl-cellulose (HPMC) (ITZ:HPMC ratio 1:1.5) produced by Janseen-Cilag [20]. The dissolution assay was performed in non-sink conditions (0.4 mg/mL of itraconazole per sample) to evaluate the release profile of the formulation in supersaturated conditions. A supersaturated drug solution has been shown to be crucial for the effective absorption of poorly water-soluble drugs [31].

**Figure 9:**
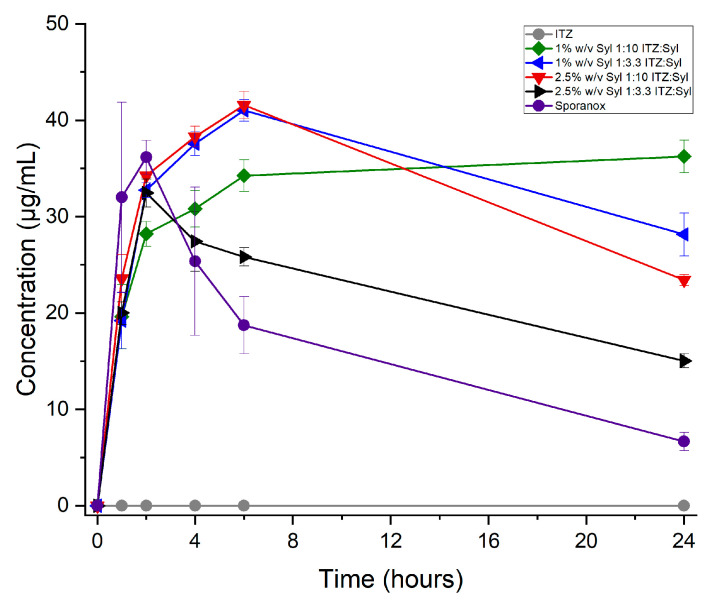
Concentration (μg/mL) released from itraconazole, electrosprayed itraconazole, Sporanox (commercially available medication) and formulations.

Crystalline unprocessed itraconazole was tested for comparison and did not show detectable dissolution under the test conditions used. As shown in Figure 9, the formulations present up to ca. 10 times enhanced release compared to itraconazole. Furthermore, the formulations show a similar or higher percentage of drug released and maintained supersaturation for longer than Sporanox. The data suggest that the drug loading concentration may play the most important role in the drug release profiles. The formulation with the lower ITZ-loading concentration, 1% *w*/*v* Syl, 1:10 ITZ:Syl, show supersaturation with sustained plateau levels under non-sink conditions for 24 h. The 1% *w*/*v* Syl, 1:3.3 ITZ:Syl, and 2.5% *w*/*v* Syl, 1:10 ITZ:Syl, which were loaded using similar itraconazole concentrations, depict sustained release reaching a peak at 6 h. Sporanox and 2.5% *w*/*v* Syl, 1:3.3 ITZ:Syl, depict a Spring and Parachute effect [32] by achieving supersaturation, peaking at 2 h, and slowly precipitating over time.

The percentage of the drug released and the actual amount released by the formulations were higher for the formulations of 1% *w*/*v* Syl 1:3.3 ITZ:Syl and 2.5% *w*/*v* Syl 1:10. The amount of itraconazole that was loaded into these formulations was comparable, as shown in Table 3.

It has been reported that the drug is released from the silica carriers in a two-step process [33,34]. The drug molecules may be physically entrapped in the MSPs or form hydrogen bonds with the hydroxyl groups on the silica surface. The physically entrapped molecules are released faster, while the molecules that have formed bonds with the MSPs release at a slower rate [33,34]. Itraconazole possesses nitrogen lone pairs and a carbonyl group that can interact with the surface of the MSPs and form hydrogen bonds [35]. An alternative explanation is that the drug molecules located on the outer surface can be released quickly (burst release), whereas the drug molecules packed in the inner pores are released more slowly [9,36,37]. The process for the second step is suggested to be as follows: the aqueous medium is absorbed into the MSPs driven by capillary force, the drug molecules dissolve into the medium inside the pores, the drug molecules diffuse out of the pores due to concentration gradient, and they further diffuse into the release medium [38]. Van Speybroeck et al. loaded 10 poorly soluble drugs with a variety of physicochemical properties in SBA-15 particles to evaluate the potential dissolution enhancement [37]. They suggested that the interactions between hydrophobic drugs and silanol groups on the silica surface were not significant in controlling the release of drugs. Instead, the rate of drug release was determined by the time required for diffusion through the internal pore network [37]. Therefore, the different drug release profiles of the formulations may be attributed to either different distributions of itraconazole on the outer surface and inner pores of the Syloid 244FP or a physical entrapment/formation of hydrogen bonds between the drug and the hydroxyl groups of the MSPs. The possibility of a combination of both processes cannot be eliminated.

It is also possible that the supersaturation led to crystal growth on the external particle surface and, thus, no further release of itraconazole from the formulations and progressively precipitation [39]. The incomplete release can be also attributed to the non-sink conditions under which the experiment was performed. Van Speybroeck et al. reported a lower release of itraconazole from an SBA-15-based formulation under non-sink conditions in comparison with sink conditions in simulated gastric fluids [20].

The incomplete release of drugs from silica particles has been reported in several other studies. It is mainly attributed to the re-adsorption of the drug on the silica [5,40], strong interactions between the drug and the hydroxyl group on the silica surface and blocked pores [5]. The pore diameter of Syloid 244FP was measured to be 19 nm, while the area covered by a molecule of itraconazole was estimated to be 2.61 nm^2^ [8]. Therefore, itraconazole should be well-deposited in Syloid 244FP pores without blocking them. Van Speybroeck et al. physically blended ITZ-loaded SBA-15 with the precipitation inhibitors hydroxypropylmethylcellulose (HPMC) and hydroxypropylmethylcellulose acetate succinate (HPMCAS) to enhance the release performance of the drug. They showed that the absence of the solubilizing agent SLS contributes to a slower and incomplete release from SBA-15. They suggested that decreased wettability and/or solubilization constitute a part of the slow and incomplete release [20]. Overall, enhanced dissolution of itraconazole was achieved from all formulations prepared using electrospraying. Also, the data suggested that drug loading concentration may play the most important role in drug release profiles.

### 3.2. ITZ-Loaded Syloid 244FP Particles Produced by Rotary Evaporation

A direct comparison between electrospraying and rotary evaporation was performed in order to evaluate the effectiveness of electrospraying to load MSPs and the impact of the technique on the solid state and the release profile of the drug. A successful outcome would be an in-depth understanding of the similarities and/or differences in the solid state and release profile of the drug from formulations produced by the two techniques and why those potential similarities and/or differences occurred. 

**Figure 10:**
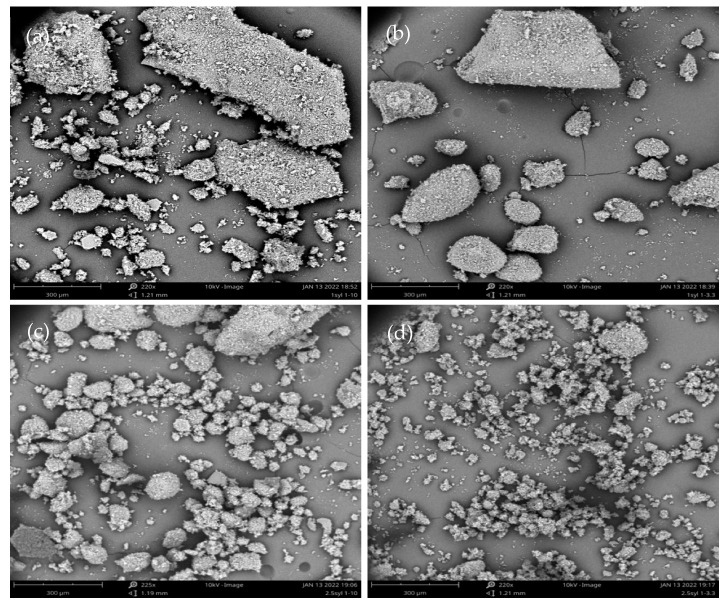
SEM images of (**a**) 1% *w*/*v* Syl 1:10 ITZ:Syl, (**b**) 1% *w*/*v* Syl 1:3.3 ITZ:Syl, (**c**) 2.5% *w*/*v* Syl 1:10 ITZ:Syl, (**d**) 2.5% *w*/*v* Syl 1:3.3 ITZ:Syl produced by rotary evaporator.

Itraconazole-loaded Syloid 244FP particles were produced using a rotary evaporator. The solid state of itraconazole in the formulations and the dissolution profile of the formulations were determined and compared with those of electrosprayed formulations. The ITZ-loaded particles prepared using a rotary evaporator appeared to be heterogeneous, coarse and large in size due to agglomeration, as shown in Figure 10. This indicates that drug loading of Syloid 244FP using a rotary evaporator resulted in aggregation. Additionally, the larger particles resemble the flat, smooth surface of pure itraconazole; thus, it is possible that itraconazole exists on the surface of the aggregates. Our findings are consistent with those of Hong et al., who used spray-drying and solvent evaporation to load fenofibrate in SBA-15 and MCM-41 at drug loadings of 30 and 50% *w*/*w*. They reported that the morphology of the MSPs was preserved after drug loading using spray-drying at both 30 and 50% *w*/*w* while, fenofibrate was observed on the external surface of the MSPs, when solvent evaporation was used at 50% *w*/*w*. They stated that the fenofibrate that existed on the external surface caused agglomeration [41].

**Figure 11:**
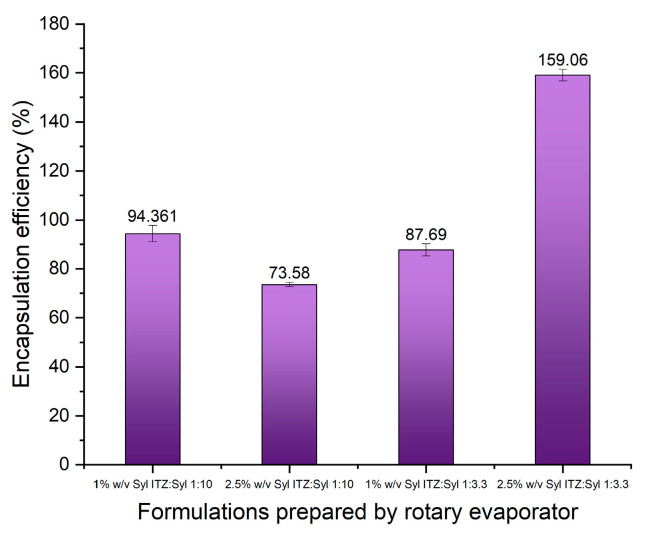
Percentage of encapsulation efficiency of 1% *w*/*v* Syl 1:10 ITZ:Syl, 0.5% *w*/*v* Syl 1:10 ITZ:Syl, 1% *w*/*v* Syl 1:3.3 ITZ:Syl, 22.5% *w*/*v* Syl 1:3.3 ITZ:Syl prepared by rotary evaporator.

The encapsulation efficiency of the formulations 1%*w*/*v* Syl 1:3.3 ITZ:Syl, 2.5% *w*/*v* Syl 1:10 ITZ:Syl and 1% *w*/*v* Syl 1:10 ITZ:Syl was high, indicating that a high percentage of itraconazole was incorporated in Syloid 244FP, as presented in Figure 11. The formulation of 2.5% *w*/*v* Syl 1:3.3 ITZ:Syl presented an EE of 159 ±2.35. After completion of the drying process in the evaporating flask, a proportion of the sample was deposited on the bottom of the flask as powder, while some needed to be scratched off the inner surface of the flask. A reasonable hypothesis may be that a part of Syloid 244FP remained on the flask walls, leading to an excessive amount of itraconazole in the formulation. It is possible that the same applied to all formulations to some extent; however, it was more evident for the formulation of 2.5% *w*/*v* Syl 1:3.3 ITZ:Syl, where the drug-loading concentration of itraconazole is higher.

**Figure 12:**
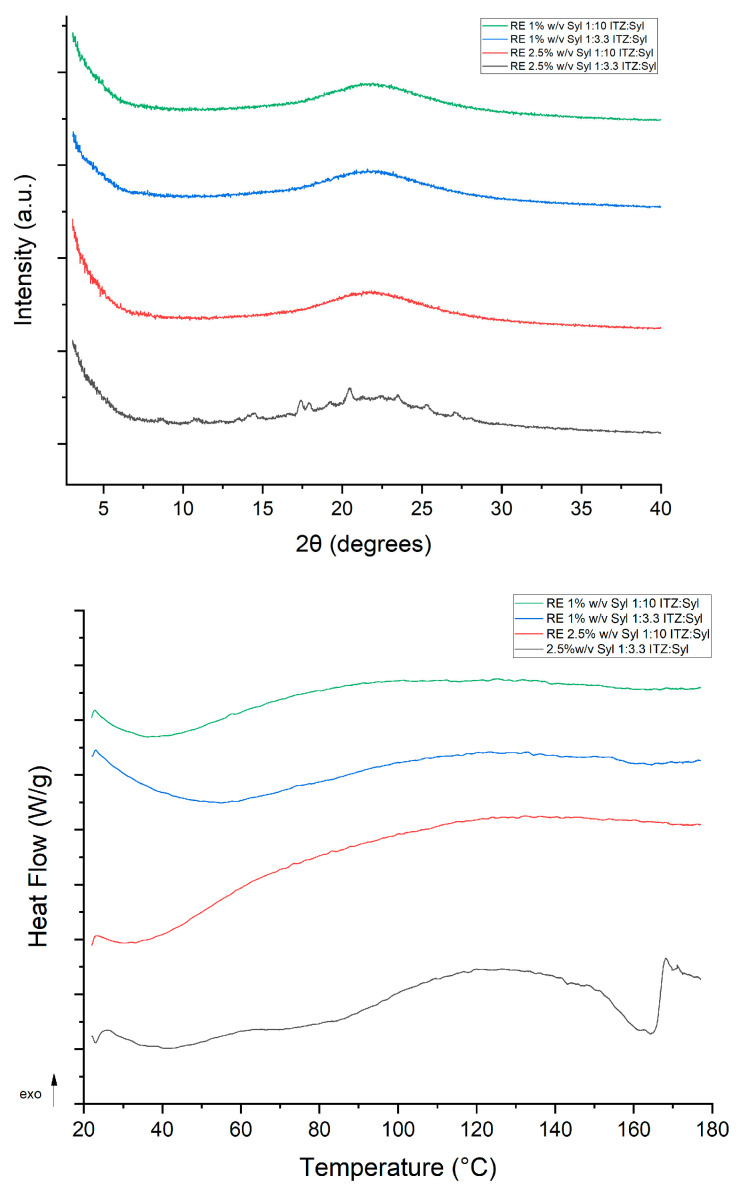
PXRD patterns (**above**) and MTDSC traces (**below**, Total Heat Flow) of itraconazole, Syloid 244FP and the formulations produced by rotary evaporator.

Most of the PXRD patterns of the formulations prepared using rotary evaporation depicted broad halos without peaks. However, the 2.5% *w*/*v* Syloid 244FP at ITZ:Syl 1:3.3 produced by a rotary evaporator demonstrated the typical PXRD peaks of itraconazole polymorph I, as demonstrated in Figure 12 (above). This suggests that itraconazole existed in a crystalline state in this formulation, which contains the highest amount of the drug. Therefore, itraconazole was present in the amorphous state in the formulation loaded using the lower drug loading concentration, while itraconazole was in the crystalline state in the formulation loaded at higher drug loading concentration. This observation is further supported by the findings of Lai et al., who utilised solvent evaporation to load ibuprofen in Syloid 244FP at various ITZ:Syl ratios (25–50% *w*/*v*) and initial drug loading concentrations. Characteristic peaks of ibuprofen were shown in all the ibuprofen-loaded Syl, indicating that crystalline ibuprofen was present. When the drug to Syloid 244FP ratio was reduced, the peaks decreased. They stated that the presence of a higher amount of Syl led to a more available area for ibuprofen to be deposited in the pores in the amorphous state [9].

The MTDSC data of the 1% *w*/*v* Syl 1:10 ITZ:Syl, 1%*w*/*v* Syl 1:3.3 ITZ:Syl and 2.5% *w*/*v* Syl 1:10 ITZ:Syl presented no endothermic transition peaks of itraconazole, suggesting the molecular dispersion of itraconazole in the formulations. Nevertheless, the 2.5% *w*/*v* Syl 1:3.3 ITZ:Syl presented two endothermic peaks at 140 and 160 °C, indicating the presence of crystalline itraconazole. A melting point in the range of 165 to 169 °C was reported for itraconazole [20].

Confined nanocrystals in mesoporous hosts were reported to display a broadening of the melting peak and depression of the melting temperature [42]. Lai et al. utilised a rotary evaporator to load Syloid 244FP with ibuprofen (IBU) at various drug loading concentrations and IBU:Syl ratios. They reported that the melting of crystalline ibuprofen inside the pores was observed at lower temperatures compared to the melting of IBU outside the pores [9]. Similar findings were reported by Marinheiro et al. who used rotary evaporation to load resveratrol in MSPs. Smaller and broader endothermic peaks were found at a lower temperature than the melting of bulk resveratrol at the DSC traces of the formulations. This suggested that part of resveratrol, which was confined to the pores, underwent crystallisation [43]. Therefore, the two endothermic peaks that are reported in this study may correspond to itraconazole on the surface and in the pores of Syloid 244FP.

**Figure 13:**
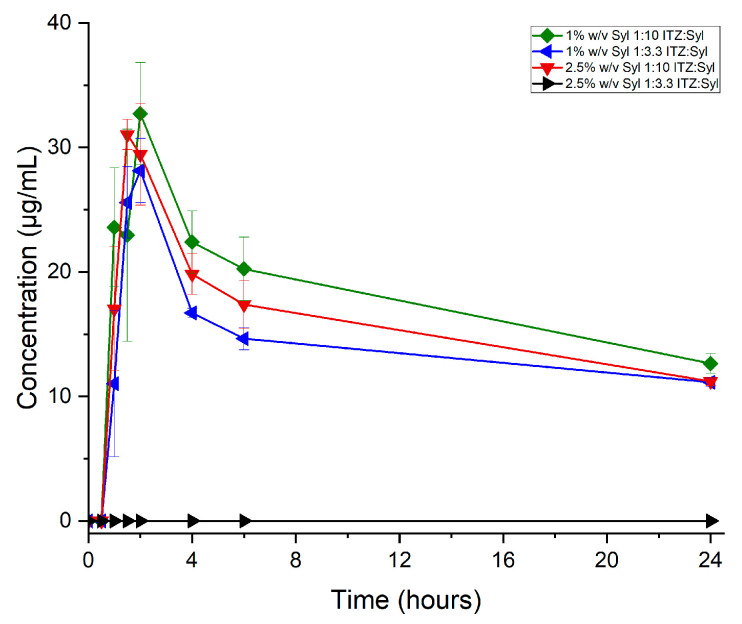
Concentration (μg/mL) released from formulations prepared using rotary evaporator.

The drug release of the formulations prepared using rotary evaporation under non-sink conditions was tested for 24 h. As shown in Figure 13, the formulations of 1% *w*/*v* Syl at two different ratios of drug to silica and the 2.5% *w*/*v* Syl 1:10 ITZ:Syl depict a burst release followed by gradual precipitation. They all reached a peak at a maximum of 2 h. The formulation of 2.5% *w*/*v* Syl 1:3.3 ITZ:Syl did not present detectable dissolution under the test conditions used. The PXRD pattern and MTDSC data suggest that crystalline itraconazole exists in this formulation, which explains the poor drug release.

The maximum percentage and amount of drug released from the formulations and the time point are shown in Table 4. The release profiles of itraconazole were enhanced for the formulations of lower drug loading concentrations produced by rotary evaporation compared to the release profile of pure itraconazole. Likewise, Lai et al. reported a faster dissolution rate and a higher percentage of drug released compared to pure ibuprofen for all formulations when they loaded Syloid 244FP with ibuprofen at different initial drug loading concentrations using rotary evaporation. Slower dissolution rates and lower supersaturation were observed for the formulation prepared at the highest initial drug loading (equivalent to the “drug loading concentration” of this study). The dissolution test was carried out under non-sink conditions in FaSSGF pH 2.0 without pepsin for 2 h [9]. The dissolution rate and supersaturation were inversely proportional to the drug loading concentration (Table S1) during the first two hours of this study, as well. However, the overall dissolution rate and supersaturation (24 h) were comparable for all formulations, where itraconazole existed in the amorphous state.

As stated, the drug release from MSPs has been suggested to be a two-step process consisting of an initial burst release followed by a slower release. The drug located on the outer surface of the pores can be released quickly, while the drug which is in the inner pores releases at a slower rate [9,36]. Alternatively, the drug that is physically entrapped leads to an initial burst release, while molecules that have formed hydrogen bonds with the hydroxyl groups on the surface of the MSPs are released at a slower rate [33,34]. However, it was observed that all formulations, apart from 2.5% *w*/*v* Syl ITZ:Syl 1:3.3 ITZ:Syl where itraconazole crystallised, presented the same one-step (only the initial burst) drug release. SEM images indicate that itraconazole was deposited on the surface of Syloid 244FP, creating agglomerates. Therefore, it is suggested that itraconazole was released mainly from the surface of Syloid 244FP, resulting in a quick initial release (supersaturation). The supersaturation led to crystal growth on the external particle surface and, thus, no further release of itraconazole from the formulations and progressively precipitation occurred [39].

### 3.3. Comparison of Electrospraying and Rotary Evaporator as Means to Produce ITZ-Loaded Syl

SEM data show that particles produced by electrospraying are more homogeneous, better dispersed and smaller in size compared to the particles produced using a rotary evaporator. This is probably due to the difference in surface area from which the evaporation occurs in the electrospraying and rotary evaporator. In electrospraying, the solvent evaporation occurs from self-dispersed droplets (micro- to nanometres in size), leading to much less agglomeration and coagulation of the particles produced. In contrast, the evaporation occurs from the surface of the flask, resulting in agglomerated particles during rotary evaporation. Hong et al. loaded fenofibrate in SBA-15 and MCM-41 using spray drying (another technique of liquid atomization) and solvent evaporation at drug loadings of 30 and 50% *w*/*w*. They reported that the morphology of the MSPs was preserved after drug loading using spray-drying at both 30 and 50% *w*/*w*, suggesting that most of the drug was incorporated in the pores. In contrast, fenofibrate was observed on the external surface of the MSPs when they were loaded using solvent evaporation at 50% *w*/*w*. They stated that the fenofibrate that existed on the external surface caused agglomeration [41]. This study supports our findings that the atomisation technique preserves the morphology of the MSPs, while the solvent evaporation leads to aggregates.

The PXRD and MTDSC results of the formulations of 1% *w*/*v* Syl 1:10, 1:3.3 ITZ:Syl and 2.5% *w*/*v* Syl 1:10 ITZ:Syl suggest that the loading technique did not affect the solid state of itraconazole when it was loaded at a concentration of up to 3.0 mg/mL. However, different drug loading techniques led to different solid states of itraconazole at the 2.5% *w*/*v* Syl 1:3.3 ITZ:Syl formulation, which was loaded at 7.5 mg/mL. Amorphous and crystalline itraconazole were present at the 2.5% *w*/*v* Syl 1:3.3 ITZ:Syl formulation produced by electrospraying and rotary evaporation, respectively. The evaporation rate of the solvent for the two loading techniques probably played a crucial role. The small volume of solvent that exists on the surface of the droplets enables fast evaporation during electrospraying, resulting in an amorphous material. In contrast, the evaporation occurs from the total volume of the suspension evaporation during rotary evaporation, leading to slower evaporation and, thus, a crystalline material. Hong et al. made similar observations and drew the same conclusions when they investigated the impact of spray-drying on the manufacturability, physiochemical stability and bioavailability of fenofibrate compared to a conventional drug loading process, namely, solvent evaporation. They reported that spray-drying enabled loading up to 50% *w*/*w* in the amorphous state of the drug onto the MSPs as opposed to the 30% *w*/*w* for solvent evaporation. They suggested that this is a result of the different solvent evaporation rates in these two methods [41]. They further proposed that the quick evaporation of a solvent through atomization in the spray-drying method appeared to be more efficient in incorporating fenofibrate in the pores, thus resulting in the amorphous state of the drug. Conversely, the solvent evaporation method probably deposited a higher amount of the drug on the surface, enabling nucleation and crystallisation due to slow evaporation during solvent removal [41]. This observation appears to also apply to our study, based on the comparative dissolution profiles.

The same formulation produced by different techniques presented different release profiles. The formulations loaded with up to 3.0 mg/mL itraconazole using electrospraying maintained the supersaturation for much longer (up to 24 h) (Figure 8 and Table 3) compared to the same formulations produced by a rotary evaporator (up to 2 h) (Figure 12 and Table 4), indicating much more robust formulations. This is probably due to the different sizes of particles, uniformity of samples and disposition of itraconazole in or on Syloid 244FP particles that each technique produces, impacting the surface area of the sample from which the drug release occurs. The findings of Mellaerts et al. [17] further support our observations. They tested the release profile of itraconazole from SBA-15 loaded using incipient wetness impregnation, solvent evaporation (using a rotary evaporator) and melt method at drug loadings of 20 and 30% *w*/*w*. The test was performed in FaSSGF with 0.5% *w*/*w* SLS. The formulations of 20 and 30% *w*/*w* prepared using wetness impregnation and solvent evaporation showed a faster release than those prepared by the melt method. They attributed the difference to a better distribution of itraconazole in the samples prepared by incipient impregnation and solvent evaporation. Moreover, they ascribed the slightly higher concentration of itraconazole released from the formulation prepared using incipient impregnation to the deposition of itraconazole in the micropore leading to a slower release [17]. Different distributions of itraconazole into the Syloid 244FP using different loading techniques are probably another reason that affected the drug release profile of the formulations. The formulation loaded at 7.5 mg/mL showed a completely different release profile when produced using electrospraying and rotary evaporation due to the different solid states in which itraconazole existed in these formulations. It is noteworthy that electrospraying enabled the production of ITZ-loaded Syl particles with different release profiles, while the rotary evaporator did not. A lack of similarity between the dissolution profiles of each formulation prepared using electrospraying and rotary evaporation (f2 < 50) is illustrated in Table S2. A summary of the comparison of electrospraying and rotary evaporator as a means to produce ITZ-loaded Syl is presented in Table 5.

Sayed et al. also highlighted the advantage of electrospraying as a loading technique for MSPs compared to solvent evaporation. They loaded mesoporous and non-porous silica particles with a chalcone synthesised by them using both electrospraying and solvent evaporation and investigated the effect of the loading techniques on the physicochemical properties and release profile of the systems produced [6]. Their data suggested that the drug was in the amorphous state in the MSPs produced by electrospraying and in crystalline state in the systems produced by rotary evaporation. They reported that the dissolution rate of the drug was faster from the electrosprayed MSPs and achieved a much higher percentage release compared to the MSPs prepared by solvent evaporation. They suggested that the significant improvement in the dissolution properties of the drug from the electrosprayed formulations was because of both the amorphization of the drug and the lower particle size that electrospraying enables [6]. Their data and interpretation are consistent with the findings of our study.

Overall, the comparison of the two techniques reveals the advantage of electrospraying over the rotary evaporation both in the physicochemical properties and release profile of ITZ-loaded Syloid 244F. This is ascribed to the better dispersion of the particles during loading/drying (atomisation), which further leads to better drug distribution in the MSPs and faster rate of evaporation due to the lower volume of the solvent and higher surface-to-volume ratio.

## 4. Conclusions

Electrospraying was successfully used as a means of loading poorly soluble drugs in MSPs in the amorphous state at high and low drug loading and MSP concentrations. Formulations prepared at different drug loading concentrations led to improved, but different among the formulations, release profiles. The different release profiles were ascribed to either the different distribution of itraconazole in the formulations or the different extent of physical entrapment and formation of hydrogen bonds between the drug and the silica surface. The data suggested that the drug loading concentration is a more important factor for the release profiles than the drug-to-carrier ratio. Comparing the two drug loading techniques, the following conclusions can be drawn. Electrospraying produced more homogeneous, better dispersed and smaller particles than rotary evaporation. Also, it was more successful in preparing amorphous and high ITZ-loaded Syloid 244FP particles, since itraconazole was in the amorphous state even at the highest drug-loaded formulation prepared by electrospraying, while it was present in the crystalline state at the highest drug-loaded formulation prepared by rotary evaporation.

Regarding the drug release profiles, the formulation of different drug loadings prepared by electrospraying showed different release profiles to each other, while the formulations of lower drug loadings prepared by a rotary evaporator demonstrated the same burst release followed by precipitation. The highest drug-loaded formulation prepared by rotary evaporation showed zero release. This is because electrospraying led to better-dispersed, smaller-in-size particles with different distributions of itraconazole in the formulations on the outer surface and inner pores of the Syloid 244FP or physical entrapment/formation of hydrogen bonds between the drug and the hydroxyl groups of the MSPs, whilst rotary evaporation led to agglomerates with itraconazole deposited on their surface. These findings are evidence that the loading technique and drug loading concentration have a leading role in the solid state and drug release profile of the formulations of poorly soluble drugs in mesoporous silica particles.

## Figures and Tables

**Table 1:** Details of ITZ concentration, ITZ:Syl ratio, voltage and flowrate of each formulation.

Formulation	ITZ Concentration	ITZ:Syl Ratio	Flowrate	Voltage
1% *w*/*v* Syl 1:10 ITZ:Syl	0.10% *w*/*v*	1:10	0.8 mL/h	18–20 kV
2.5% *w*/*v* Syl 1:10 ITZ:Syl	0.25% *w*/*v*	1:10	0.8 mL/h	20–21 kV
1% *w*/*v* Syl 1:3.3 ITZ:Syl	0.30% *w*/*v*	1:3.3	0.8 mL/h	16.6–17.6 kV
2.5% *w*/*v* Syl 1:3.3 ITZ:Syl	0.75% *w*/*v*	1:3.3	0.8 mL/h	15–16 kV

**Table 2:** Surface area, pore volume and size of Syloid 244FP and formulations prepared by electrospraying.

Samples	Surface Area (m^2^/g)	Pore Volume (cm^3^/g)
Syloid 244FP	364.7	1.74
1% *w*/*v* Syl ITZ:Syl 1:10	91.7	0.591
1% *w*/*v* Syl ITZ:Syl 1:3.3	44.1	0.243
2.5% *w*/*v* Syl ITZ:Syl 1:10	142.9	0.708
2.5% *w*/*v* Syl ITZ:Syl 1:3.3	167.0	0.826

**Table 3:** Maximum drug released (cumulative percentage and amount) at the time achieved from the formulations prepared by electrospraying.

Samples	ITZ Concentration in Solution	Drug Release %	Amount Released (mg)
1% *w*/*v* Syl ITZ:Syl 1:10	10mg/mL	9.01 ± 0.42	0.36 ± 0.02
2.5% *w*/*v* Syl ITZ:Syl 1:10	25 mg/mL	10.34 ± 0.35	0.41 ± 0.01
1% *w*/*v* Syl ITZ:Syl 1:3.3	30 mg/mL	10.33 ± 0.28	0.41 ± 0.01
2.5% *w*/*v* Syl ITZ:Syl 1:3.3	75 mg/mL	8.17 ± 0.37	0.32 ± 0.01

**Table 4:** Maximum drug released (percentage and amount) at the time achieved from the formulations prepared by a rotary evaporator.

Samples	Drug Release %	Amount Released (mg)	Time (h)
1% *w*/*v* Syl ITZ:Syl 1:10	8.139 ± 1.021	0.327 ± 0.041	2
2.5% *w*/*v* Syl ITZ:Syl 1:10	7.72 ± 0.31	0.310 ± 0.012	1.5
1% *w*/*v* Syl ITZ:Syl 1:3.3	7.08 ± 1.021	0.310 ± 0.012	2
2.5% *w*/*v* Syl ITZ:Syl 1:3.3	0	0	0

**Table 5:** Comparison of electrospraying and rotary evaporator as a means to produce ITZ-loaded Syl.

	Electrospraying	Rotary Evaporation
SEM and particle size	Relatively homogeneous, well dispersed, submicron size range up to 8 μm	Heterogeneous, coarse and agglomerations in the scale of tens to hundreds of μm
Encapsulation Efficiency	91–118% (refer to Section 3.1)	73–159% (refer to Section 3.2)
Solid state (PXRD, MTDSC) of itraconazole in samples loaded at a concentration of up to 3.0 mg/mL	Amorphous	Amorphous
Solid state (PXRD, MTDSC) of itraconazole in samples loaded at a concentration up to 7.5 mg/mL	Amorphous	Crystalline
Dissolution profiles of formulations loaded with up to 3.0 mg/mL itraconazole	Maintained the supersaturation for up to 24 h	Maintained the supersaturation for up to 2 h
Dissolution profiles of formulations loaded with up to 7.5 mg/mL itraconazole	Maintained the supersaturation for up to 2 h	No detectable dissolution

## Data Availability

Data are contained within the article and Supplementary Materials.

## Supplementary Materials

The following supporting information can be downloaded at: https://www.mdpi.com/article/10.3390/pharmaceutics16081102/s1, Figure S1: MTDSC traces of physical mixture of itraconazole: Syloid 244FP 1:3 (above) and 1:10 below; Table S1: Drug loading concentrations used for each formulation ; Table S2: Values for similarity factor (f2) of the release profiles of each formulation prepared using electrospraying and rotary evaporation
