# Supplementary material for: Electrospraying as a Means of Loading Itraconazole into Mesoporous Silica for Enhanced Dissolution
Source: Pharmaceutics. 2024 Aug 22;16(8):1102. doi: 10.3390/pharmaceutics16081102 (PMC11359385; doi:10.3390/pharmaceutics16081102)
Supplement: Supplementary file 1 [file pharmaceutics-16-01102-s001.zip › pharmaceutics-3099951-supplementary.pdf]

Electronic Supplementary Information

Electrospraying as a Means of Loading Itraconazole into Mesoporous Silica for Enhanced Dissolution

Charitini Volitaki[1]; Andrew Lewis[2]; Duncan Q.M. Craig [3]; Asma Buanz[1,4*]

[1]UCL School of Pharmacy, 29-39 Brunswick Square, London, WC1N 1AX, UK
[2] Quotient Sciences, Mere Way, Ruddington, Nottingham, NG11 6JS, UK
[3] Faculty of Life Sciences, University of Bath, Claverton Down, Bath, BA2 7AY, UK; dqmc21@bath.ac.uk
[4] School of Science, Gillingham, Medway, ME4 4TB, UK
* Correspondence: a.buanz@gre.ac.uk

Table S1: Drug loading concentrations used for each formulation

Samples	ITZ-loading concentration
**1% w/v Syl 1:10 ITZ:Syl**	1.00 mg/mL
**2.5% w/v Syl 1:10 ITZ:Syl**	2.50 mg/mL
**1% w/v Syl 1:3.3 ITZ:Syl**	3.00 mg/mL
**2.5% w/v Syl 1:3.3 ITZ:Syl**	7.50 mg/mL

Table S2: Values for similarity factor (f2) of the release profiles of each formulation prepared using electrospraying and rotary evaporation

Samples	f2
**1% w/v Syl 1:10 ITZ:Syl**	44.05
**1% w/v Syl 1:3.3 ITZ:Syl**	37.96
**2.5% w/v Syl 1:10 ITZ:Syl**	40.99
**2.5% w/v Syl 1:3.3 ITZ:Syl**	30.18

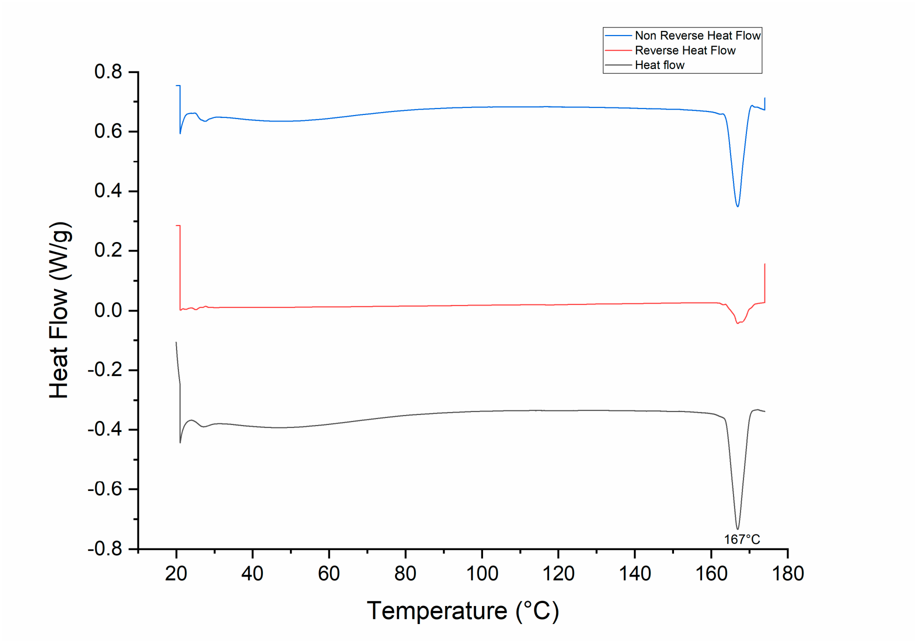

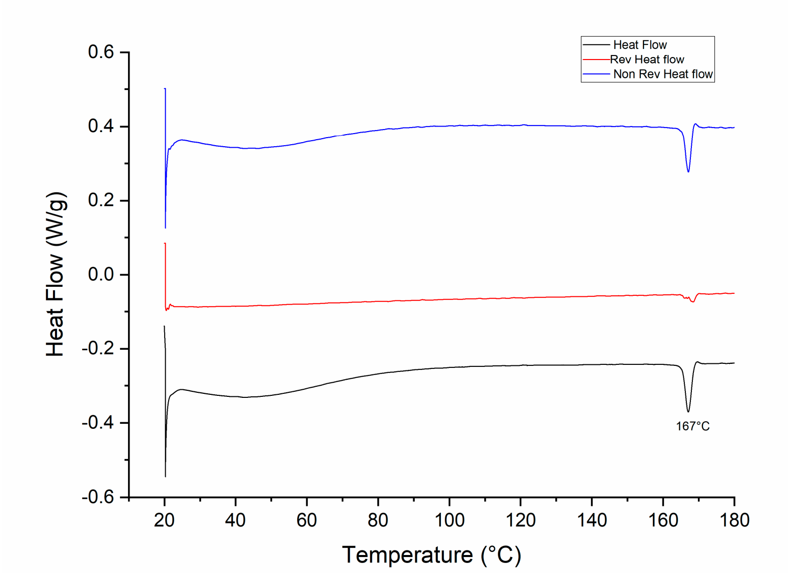

Figure S1: MTDSC traces of physical mixture of itraconazole: Syloid 244FP 1:3 (above) and 1:10 below
